# ChatGPT-4 and the Global Burden of Disease Study: Advancing Personalized Healthcare Through Artificial Intelligence in Clinical and Translational Medicine

**DOI:** 10.7759/cureus.39384

**Published:** 2023-05-23

**Authors:** Mohamad-Hani Temsah, Amr Jamal, Fadi Aljamaan, Jaffar A Al-Tawfiq, Ayman Al-Eyadhy

**Affiliations:** 1 Pediatric Intensive Care Unit, Department of Pediatrics, King Saud University Medical City, College of Medicine, King Saud University, Riyadh, SAU; 2 Department of Family and Community Medicine, College of Medicine, King Saud University, Riyadh, SAU; 3 Evidence-Based Health Care & Knowledge Translation Research Chair, Department of Family and Community Medicine, College of Medicine, King Saud University, Riyadh, SAU; 4 Department of Critical Care, College of Medicine, King Saud University, Riyadh, SAU; 5 Department of Specialty Internal Medicine and Quality, Johns Hopkins Aramco Healthcare, Dhahran, SAU; 6 Infectious Disease Division, Department of Medicine, Indiana University School of Medicine, Indianapolis, USA; 7 Infectious Disease Division, Department of Medicine, Johns Hopkins University School of Medicine, Baltimore, USA; 8 Pediatric Intensive Care Unit, Department of Pediatrics, College of Medicine, King Saud University, Riyadh, SAU; 9 Pediatric Intensive Care Unit, King Saud University Medical City, Riyadh, SAU

**Keywords:** precision medicine, personalized healthcare plan, chatbots, ai-assisted personalized disease burden, chatgpt-4, global burden of disease (gbd)

## Abstract

The fusion of insights from the comprehensive global burden of disease (GBD) study and the advanced artificial intelligence of open artificial intelligence (AI) chat generative pre-trained transformer version 4 (ChatGPT-4) brings the potential to transform personalized healthcare planning. By integrating the data-driven findings of the GBD study with the powerful conversational capabilities of ChatGPT-4, healthcare professionals can devise customized healthcare plans that are adapted to patients' lifestyles and preferences. We propose that this innovative partnership can lead to the creation of a novel AI-assisted personalized disease burden (AI-PDB) assessment and planning tool.

For the successful implementation of this unconventional technology, it is crucial to ensure continuous and accurate updates, expert supervision, and address potential biases and limitations. Healthcare professionals and stakeholders should have a balanced and dynamic approach, emphasizing interdisciplinary collaborations, data accuracy, transparency, ethical compliance, and ongoing training.

By investing in the unique strengths of both ChatGPT-4, especially its newly introduced features such as live internet browsing or plugins, and the GBD study, we may enhance personalized healthcare planning. This innovative approach has the potential to improve patient outcomes and optimize resource utilization, as well as pave the way for the worldwide implementation of precision medicine, thereby revolutionizing the existing healthcare landscape. However, to fully harness these benefits at both the global and individual levels, further research and development are warranted. This will ensure that we effectively tap into the potential of this synergy, bringing societies closer to a future where personalized healthcare is the norm rather than the exception.

## Editorial

The global burden of disease (GBD) study exemplifies big-data usage in healthcare, while open artificial intelligence (AI) chat generative pre-trained transformer latest version 4 (ChatGPT-4) is a rapidly emerging artificial intelligence (AI)-chatbot, with variable potential applications [1,2]. Though the GBD provides global health insights, its broad scope limits its application in personalized medical plans. In contrast, ChatGPT-4 quickly processes extensive information, but its accuracy relies on its training data. Integrating both systems may facilitate more prompt, individualized, and data-driven decisions by healthcare professionals [3].

**Figure 1:**
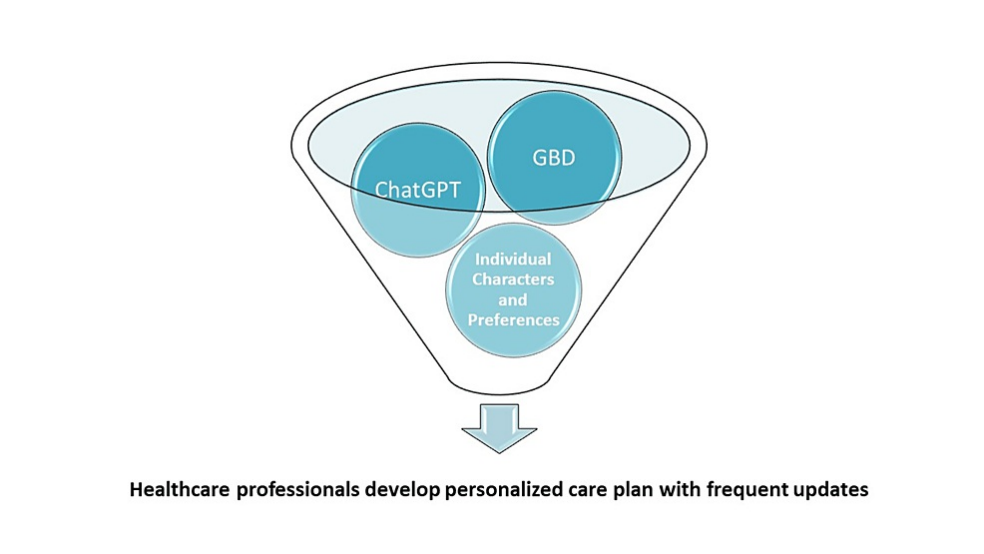
AI-assisted personalized disease burden (AI-PDB) suggested model. Suggested model for empowering healthcare professionals with ChatGPT-4 to integrate the most recent GBD study results with the individual’s characters and personal preferences to formulate her/his AI-PDB personalized healthcare plans. AI: artificial intelligence, ChatGPT-4: chat generative pre-trained transformer version 4, GBD: global burden of disease.

We suggest a model where healthcare professionals utilize ChatGPT-4 to combine GBD insights with individual characteristics, such as lifestyle and personal preferences, for unique personalized healthcare plans (Figure 1). This integration could evolve into an AI-assisted personalized disease burden (AI-PDB) assessment and planning tool. The model must be frequently updated based on patient needs and medical literature, with expert oversight to ensure accuracy and address potential biases in the generated plan [4,5].

For the successful integration of data-driven GBD insights and the user-friendly AI chatbot ChatGPT-4, healthcare professionals and stakeholders must adopt a collaborative, balanced, and dynamic approach [3]. This involves incorporating both resources into individualized care while critically and ethically evaluating the created output. Recognizing each system's strengths and limitations will result in a better foundation for personalized medical decisions and specific healthcare recommendations.

Healthcare professionals should remain vigilant for potential AI chatbot drawbacks, such as data privacy, text accuracy, and difficulties capturing individual nuances compared to clinical judgment. Conversely, big data issues like interoperability, inconsistent definitions, and selection bias require attention [3]. To refine healthcare big data and interactive tools, continued investment is essential [1,3]. This includes promoting interdisciplinary collaborations, ensuring data accuracy, transparency, ethical compliance, adherence to legal standards, equitable usage, and providing ongoing training for professionals to effectively utilize these resources.

**Figure 2:**
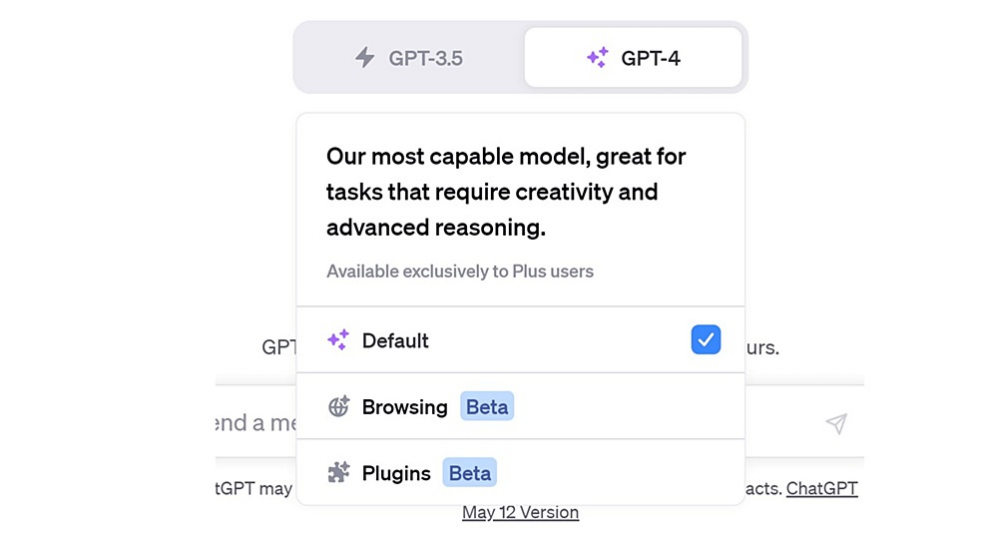
Screenshot of ChatGPT-4 new beta versions of internet browsing or plugins.

As we look towards the future of healthcare, we propose further research into the integration of AI chatbots, particularly the newly introduced features of ChatGPT-4 such as live internet browsing or plugins (Figure 2). These advancements could facilitate a more seamless interaction with the invaluable insights from the GBD study. This integration holds the potential to unlock untapped possibilities and unveil new opportunities for personalized healthcare planning. By facilitating rapid and tailored decision-making, such an approach could enhance patient outcomes and optimize resource allocation within the healthcare system. It is crucial, however, to acknowledge the limitations of each model and prioritize collaborative interactions between healthcare professionals and policymakers. One possibility is to have AI chatbot access to regional data from GBD and integrate this data into a risk-based assessment of specific diseases, potentially formulating region-based recommendations for the prevention and control of locally prevalent diseases or conditions.

In conclusion, we emphasize the need for continued research and innovation to explore various types of integration between AI chatbots and GBD, and to refine the provision of personalized healthcare through the utilization of AI and big data. Another possibility is to have an AI chatbot utilize the data from GBD to extrapolate the impact of population-based interventions on the reduction of the most common diseases/conditions. The ultimate goal is to achieve precision medicine for all individuals and enhance global health outcomes. To this end, increased investment in this domain is vital. By prioritizing this area of research, we can move closer to a future where personalized patient care is not just a possibility, but a reality.
